# Self-management behavior, associated factors and its relationship with social support and health literacy in patients with obstructive sleep apnea–hypopnea syndrome

**DOI:** 10.1186/s12890-022-02153-1

**Published:** 2022-09-18

**Authors:** Haitao Yu, Ye Gao, Tong Tong, Chunguang Liang, Hui Zhang, Xiangru Yan, Liying Wang, Huiying Zhang, Hongliang Dai, Huijuan Tong

**Affiliations:** 1 Department of Nursing, School of Nursing, Jinzhou Medical University, No 40, Section 3, Songpo Road, Jinzhou, 121001 China; 2 Liaoning Vocational University of Technology, Jinzhou, China; 3 The First Affiliated Hospital, Jinzhou Medical University, Jinzhou, China; 4 School of Nursing, Shenyang Medical College School, Shenyang, China

**Keywords:** Obstructive sleep apnea–hypopnea syndrome, Self-management behavior, Social support, Health literacy

## Abstract

**Background:**

The proportion of patients with obstructive sleep apnea–hypopnea syndrome (OSAHS) is increasing year by year in China, which has become a major public health problem. Self-management of OSAHS and multiple support from caregivers are key to low hospital admissions and high quality of life for patients with OSAHS. Social support and health literacy are the main promoters of self-management behavior. However, their contributions have not been adequately studied. The purpose of this study is to investigate the level of self-management among patients with OSAHS and its relationship with general demographics, social support, and health literacy.

**Methods:**

A total of 280 patients with OSAHS treated in two Classiii Grade A hospitals in Jinzhou City, Liaoning Province from October 2020 to July 2021 were selected as the study subjects. Patients were investigated by General Characteristics Questionnaire, Social Support Rating Scale (SSRS), Health Literacy Scale for Chronic Patients (HLSCP), and OSAHS Self-management Behavior Questionnaire, and the influencing factors of self-management of patients with OSAHS were analyzed.

**Results:**

The average score of OSAHS self-management was 74.49(SD = 8.06), SSRS and HLSCP scores were positively correlated with total scores of self-management behavior. Furthermore, we found that disease duration, SSRS, and HLSCP scores were the main predictors of self-management behavior (R^2^ = 0.390, *P* < 0.001).

**Conclusion:**

This study found that OSAHS patients with a longer duration of disease and higher SSRS or HLSCP scores also had higher levels of self-management. The factors discussed in this study may be helpful in developing individualized interventions in self-management for patients with OSAHS.

## Background

Obstructive sleep apnea-hypopnea syndrome (OSAHS) is a chronic condition in which the upper airway collapses during sleep causing obstruction, resulting in apnoea, hypoventilation, hypercapnia, and hypoxemia [1]. According to statistics, nearly one billion people worldwide have OSAHS, of which China has the largest number of OSAHS patients in the world, with over 176 million (23.6%) at high risk of OSAHS in the 30–69 age group [2]. The incidence of OSAHS is increasing every year and has become a public problem that endangers human health [3]. Effective self-management behaviors can reduce OSAHS-related risk factors, decrease the occurrence of complications and delay the development of OSAHS [4, 5]. However, low awareness of the importance of self-management and lack of adherence among OSAHS patients has been reported [6]. Therefore, this study needs to identify potential predictors of patient self-management.

Self-management is a health behavior in which individuals continually treat their illness by maintaining and improving their health and reducing the impact of their illness on their social roles, ideology, emotional responses, and relationships [7]. The recurrence of OSAHS is closely related to negative emotional states and bad behavior patterns, leading to unimaginable consequences [8]. Social support as a protective factor has been widely used in the field of chronic diseases and has attracted attention. In addition, relevant studies have proven that a good social support system is conducive to alleviating patients' negative psychological states and has a positive effect on improving patients' self-management [9, 10]. Health literacy, as an important indicator to evaluate an individual's ability to access and apply health information, is important in improving the physical emotional, and psychological well-being of patients [11].

Social support can be obtained from relatives, friends, and social groups, and is an interpersonal resource that can be accessed and used when dealing with life's challenges and stresses [12]. The previous study has shown that social support such as sleep health training for black peers with OSAHS can improve treatment adherence in people with OSAHS [13]. Patients with higher levels of social support are more able to cope with their environment and life changes, and the burden on their primary family carers is relatively less [14]. OSAHS patients with good social support systems can achieve better treatment outcomes and quality of life through long-term progressive self-management behaviors compared to those with poor social support [15].

Health literacy is the ability of an individual to acquire, understand and process the relevant knowledge and information about health services needed for disease and to make sound decisions [11]. A study of functional health literacy in people with OSAHS found that levels of health literacy are important contributors to individuals' participation in health decision-making, coping with complex health systems, and improving the quality of health services [16]. Another study emphasizes that the health knowledge of patients should be considered in the diagnosis and treatment of OSAHS patients [17]. People with high health literacy are more likely to have accurate general knowledge of diseases and to screen and access health information, thus contributing to the self-management of diseases [18].

In summary, social support and health literacy may be strongly associated with self-management behaviors. However, it is unclear whether social support and health literacy lead to better self-management behaviors in OSAHS patients. Therefore, this study aimed to investigate the level of self-management in OSAHS patients and its relationship with socio-demographic factors, social support, and health literacy.

## Methods

### Participants

This study investigated 280 patients with OSAHS in two hospitals in Jinzhou City, Liaoning Province from October 2020 to July 2021. Patients must meet the following criteria: (1) Patients with an apnea–hypopnea index (AHI) ≥ 5 h and diagnosed with OSAHS by PSG were included in the study; (2) Age ≥ 18 years; (3) No CPAP or surgery; (4) Informed consent and voluntary participation in this study. Exclusion criteria: (1) patients with severe organ dysfunction such as heart, brain, and kidney; (2) patients with severe visual impairment, hearing abnormalities, and other factors that prevent them from completing the questionnaire; (3) patients with cognitive impairment and mental disorders. Before testing, the purpose and significance of the survey are explained in detail to the respondent and informed consent is obtained by signing an informed consent form. The study plan had been approved by the Ethics Committee of Jinzhou Medical University (Ethics Approval Number: LLSC2020008).

### Polysomnography

All patients underwent nocturnal polysomnography monitoring and were recorded for more than 7 h. The monitoring program includes multi-channel physiological signals such as EEG, EOG, EMG, pulse oximetry, chest and abdominal airflow movements, and body movements of the subjects. The recorded data were interpreted according to the guidelines of the American Academy of Sleep Medicine (AASM). Sleep apnoea is defined as the absence or significant reduction (≥ 90% from baseline) in nasal and oral airflow during sleep, lasting ≥ 10 s. Hypopnea was defined as a 30% reduction in the airflow for at least 10 s with > 3% oxygen desaturation. The AHI is defined as the average number of obstructive apnoea and hypoventilation events per hour of sleep and is an objective indicator of the severity of OSAHS. According to the AHI, OSAHS can be classified as mild (5 ≤ AHI < 15/h), moderate (15 ≤ AHI < 30/h), and severe (AHI ≥ 30/h).

### Instrument

#### Social support rating scale (SSRS)

Compiled by Xiao [19] in 1986, it includes three dimensions: objective support, subjective support, and utilization of support. Each dimension contains corresponding items: 2, 6 and 7 items, 1, 3–5 and 8–10 items. The scale includes single-choice and multiple-choice items, and the score ranges from 12 to 66. Higher scores indicate that the individual receives, feels, and uses social support to a better degree. The scale is widely used to assess social support in healthy people, chronic diseases, and other research areas. In this study, the overall Cronbach's α scale was 0.730, which has good reliability.

#### Health literacy scale for chronic patients (HLSCP)

The scale was developed by Jordan JE, Australia, in 2010 to measure and evaluate individual health literacy [20]. Later translated and locally revised by Sun et al. [21] in 2012, it has 24 items in total, covering information acquisition ability, communication interaction ability, health improvement willingness, and economic support willingness. The sum of each item represents an individual's health literacy status. Higher scores indicate higher levels of health literacy. Scores above 96 are considered to have chronic health literacy. The scale has good psychometric indicators, and it is widely used in patients with chronic diseases. In this study, the overall Cronbach's α of the scale was 0.890, which has good reliability.

#### OSAHS self-management behavior questionnaire

The OSAHS Self-management Behavior Questionnaire was developed by Dong et al. [22] in 2014, which contains four dimensions, namely life management, treatment management, knowledge and skill management, and psychological management, with a total of 21 items. The scores of these 21 items range from 1 to 5, and the sum of each item represents the individual's self-management status, with higher total scores representing higher levels of self-management. Among them, the total score of self-management is divided into three grades: general (total score ≤ 63), medium (total score 63–84), and good (total score ≥ 84). In this study, the overall Cronbach's α of the scale was 0.856, which has good reliability.

### Demographic characteristic

General Characteristics Questionnaire included age, sex, living area, education level, marital status, per capita monthly income, disease duration, medical insurance, family history of snoring, and health problem other than OSAHS.

### Statistical analysis

SPSS 25.0 statistical software was used for statistical analysis. Measurement data were expressed as mean ± standard deviation (Mean ± SD), and counting data were expressed as (n, %). T-test or one-way ANOVA was used for the mean test and multiple linear regression was used to analyze the influencing factors of self-management, *P* < 0.05 was considered statistically significant.

## Results

### Baseline characteristics of OSAHS

Among the 280 OSAHS patients included in this study, 78.6% were male and 21.4% were female. The age distribution of the subjects was mostly 31–50 years old, with an average age of 44.56 ± 9.89. In this study, 94.3% of patients had a spouse. 71.4% of the patients lived in urban areas and 28.6% in rural areas. The educational level of the participants was junior high school (32.5%) and 75.0% of the participants had a monthly income of more than 3,000 RMB (1 RMB = 0.15 USD). Majority of patients with severe OSAHS (47.9%). 72.9% of the patients had medical insurance. 65.0% of the patients had a family history of snoring. There were 156 patients (55.7%) with a disease course of fewer than 5 years and 134 patients (47.9%) with health problem other than OSAHS (Table 1).

**Table 1:** Sample characteristics (n = 280, %)

Characteristics	N	%
*Sex*		
Male	220	78.6
Female	60	21.4
*Age (years)*		
≤ 30	21	7.5
31–50	169	60.4
≥ 51	90	32.1
*Marital status*		
Married	264	94.3
Single/divorced/widowed	16	5.7
*Living area*		
City	200	71.4
Rural	80	28.6
*Level of education*		
Primary and below	81	28.9
Junior high school	91	32.5
High school	82	29.3
Junior college or above	26	9.3
Monthly income(RMB)*		
< 1000	20	7.1
1000–3000	50	17.9
> 3000	210	75.0
*Severity of OSAHS*		
Mild	24	8.6
Moderate	122	43.6
Severe	134	47.9
*Disease duration (years)*		
< 5	156	55.7
≥ 5	124	44.3
*Medical insurance*		
Yes	204	72.9
No	76	27.1
*Family history of snoring*		
Yes	182	65.0
No	98	35.0
*Health problem other than OSAHS*		
Yes^a^	134	47.9
No	146	52.1

*1 RMB = 0.15 USD

^a^Any one of these diseases: Hypertension, diabetes, chronic obstructive pulmonary disease, atrial fibrillation, hyperlipidemia, depression, etc.

### Factors associated with self-management behavior

Table 2 shows the association between self-management of OSAHS patients and the general demographic characteristics of study participants. General demographic characteristics associated with self-management included living area (t = 2.704, *P* = 0.007), education level (F = 10.105, *P* = 0.000), monthly income (F = 13.123, *P* = 0.000), disease duration (t = − 2.777, *P* = 0.006), family history of snoring (t = − 2.780, *P* = 0.006), and health problem other than OSAHS (t = 3.061, *P* = 0.002).

**Table 2:** Comparison of self-management level of OSAHS patients with different characteristics (n = 280, Mean ± SD)

Characteristics	Self-management scores		
	Mean ± SD	t or F	*P* value
*Sex*			
Male	74.50 ± 8.36	0.075	0.940
Female	74.42 ± 6.90		
*Age (years)*			
≤ 30	73.57 ± 1.41	0.157	0.854
31–50	74.50 ± 8.10		
≥ 51	74.67 ± 8.38		
*Marital status*			
Married	74.47 ± 8.14	0.135	0.893
Single/divorced/widowed	74.75 ± 6.67		
*Living area*			
City	75.30 ± 7.86	2.704	0.007
Rural	72.45 ± 8.22		
*Level of education*			
Primary and below	71.73 ± 7.93	10.105	0.000
Junior high school	74.38 ± 7.88		
High school	75.23 ± 7.31		
Junior college or above	81.08 ± 7.40		
*Monthly income(RMB)*			
< 1000	66.15 ± 9.82	13.123	0.000
1000–3000	74.06 ± 6.16		
> 3000	75.38 ± 7.84		
*Severity of OSAHS*			
Mild	76.33 ± 6.64	0.904	0.406
Moderate	73.98 ± 8.30		
Severe	74.63 ± 8.06		
*Disease duration (years)*			
< 5	73.31 ± 8.15	− 2.777	0.006
≥ 5	75.97 ± 7.72		
*Medical insurance*			
Yes	75.04 ± 8.07	− 1.909	0.057
No	72.97 ± 7.87		
*Family history of snoring*			
Yes	75.46 ± 7.45	− 2.780	0.006
No	72.68 ± 8.35		
*Health problem other than OSAHS*			
Yes	72.97 ± 7.44	3.061	0.002
No	75.88 ± 8.37		

### Correlation between SSRS, HLSCP, and self-management in OSAHS patients

The average scores of SSRS, HLSCP, and self-management behavior of OSAHS patients and their correlation are shown in Table 3. Correlation analysis showed that there was a positive correlation between SSRS and HLSCP (r = 0.350, *P* < 0.05), SSRS was positively correlated with self-management (r = 0.340, *P* < 0.05), HLSCP was positively correlated with self-management (r = 0.583, *P* < 0.05).

**Table 3:** Correlation between SSRS, HLSCP, and Self-management in OSAHS patients (n = 280, Mean ± SD)

Variables	Mean ± SD	SSRS	HLSCP
		r (*p*-value)	r (*p*-value)
SSRS	40.53 ± 5.15	1	
HLSCP	88.06 ± 9.98	0.350**	1
Self-management behavior	74.49 ± 8.06	0.340**	0.583**

*SSRS* social support rating scale, *HLSCP* health literacy scale for chronic patients

**p < 0.01

### Predictors of self-management behavior

To explore the main factors influencing the self-management level of OSAHS patients. The total score of self-management was set as a dependent variable, social support score, health literacy score, and factors with statistical significance in single factor analysis (living area, education level, per capita monthly income, disease duration, family history of snoring, and health problem other than OSAHS) were taken as independent variables. Multiple linear regression analysis showed that the duration of disease, social support, and health literacy had a significant effect on self-management, as shown in Table 4.

**Table 4:** Multiple linear regression analysis of self-management in OSAHS patients

Variables	B	SE	β	t	*p*
(Constant)	24.417	5.574	–	4.381	0.000
Disease duration	1.600	0.786	0.099	2.036	0.043
SSRS	0.246	0.087	0.158	2.840	0.005
HLSCP	0.421	0.044	0.521	9.664	0.000

*SSRS* social support rating scale, *HLSCP* health literacy scale for chronic patients

## Discussion

This study examines the level of self-management of patients with OSAHS and its relationship with general demographic characteristics, SSRS, and HLSCP. Compared to other chronic diseases, self-management behaviors of OSAHS have been less studied.

Our study found limited self-management in patients with OSAHS, with only 13.9% of patients having a good level of self-management. According to the study [23], over 80% of people at risk of OSAHS are undiagnosed and untreated due to a lack of awareness of OSAHS. The low cognitive level is an important cause of poor self-management behavior in patients [24]. In a study in rural Nepal, practitioner and patient-related factors were also cited as reasons for the limited level of self-management [25]. For example, most physicians focused only on clinical outcomes and neglected the critical role of self-management in improving disease outcomes and quality of life. In addition, many patients feel that the information support provided by health care professionals does not match their wishes, leading to limitations in self-management.

This study found significant differences in the level of self-management among OSAHS patients with different demographic characteristics. The higher level of self-management among OSAHS patients living in cities may be related to easier and more convenient access to medical knowledge and resources for urban residents [26]. The finding that higher educational attainment was associated with better levels of self-management in our study is supported by other research which has identified higher educational attainment as a significant predictor of improved self-management behavior [22, 27]. A study by Tarasiuk et al. found that financial subsidies can lead to more positive treatment intentions and better healthcare experiences in patients with OSAHS, suggesting that financial conditions are an important factor influencing patients' willingness to treat and receive treatment promptly [28]. Patients from well-off families are better able to afford treatment, helping them to better manage their disease and slow its progression. The longer the course of the disease, the higher the level of self-management, which is consistent with the findings in hypertensive patients [29]. However, different from the study on COPD patients, it may be related to individual differences in samples [30]. We found that patients with a family history of snoring had better OSAHS self-care. These findings confirmed that family members play an active role in identifying disease symptoms and monitoring medical behaviors [31]. People with no health problems other than OSAHS have a higher level of self-care. It may be that managing one's health is more difficult when the patient has multiple health problems [32].

This study also found a positive correlation between SSRS, HLSCP, and total self-management scores. No previous studies have been conducted to correlate SSRS, HLSCP, and self-management in OSAHS patients. However, the association between these potential variables has been investigated in other areas of chronic disease. Consistent with our results, Chen et al. [33] studied patients with chronic kidney disease and showed that self-management behaviors were associated with patients' social support as well as health literacy. This was also a consistent finding in a study of patients with heart failure [10]. Early studies have also shown that SSRS [34, 35] and HLSCP [32, 36] are positively associated with self-management behavior, respectively.

Regression analysis showed that disease duration, SSRS, and HLSCP scores were the main predictors of self-management behavior. This is consistent with a study on another chronic disease [33], showing that social support has a positive predictive effect on self-management. The level of social support is thought to influence an individual's internal perceptions, with positive social interactions increasing positive self-perceptions [37]. For example, encouragement and support from partners are important for people with OSAHS to adhere to and implement health management plans (weight loss, smoking cessation, alcohol cessation) [38]. With the valuable advice and substantial help of others, people with OSAHS can receive more information and supervision, which will strengthen their resolve to overcome difficulties and solve problems, helping patients to better manage their disease [39, 40]. In this study, health literacy was also a significant predictor of self-care behavior in patients with OSAHS. Reisi et al. identified that the level of health literacy plays an essential role in the acquisition of disease-related knowledge by individuals and in the use of the knowledge gained to promote and maintain physical health [41]. In addition, when patients acquire a certain level of health knowledge, their self-management will subsequently improve.

The severity of OSAHS is a major contributor to poor quality of life, poor health, and high social costs, so improving the self-management of people with OSAHS is particularly crucial [42]. Studies have confirmed that self-management interventions for people with OSAHS, such as a sensible, regular diet plan and exercise program, can reduce BMI, improve AHI and reduce the risk of disease [43, 44]. The above findings suggest that patients with OSAHS should be aware of the need to actively learn effective disease management practices. In addition, relatives, friends, and social groups around OSAHS patients should provide more support to improve their self-management ability. At the same time, caregivers should be aware that they have a vital role to play in educating patients on the correct use of positive pressure ventilation and ensuring a balance between lifestyle interventions [45]. Furthermore, nursing staff can further strengthen patients' self-management skills and develop effective clinical care pathways based on individual differences to improve the overall health of patients with OSAHS.

This study has some limitations. First, the sample was obtained and selected by a single method, which may lack representativeness. In addition, this study was only conducted in Jinzhou City, Liaoning Province. Therefore, our results should be generalized with caution. Second, because of the self-reported nature of the survey, potential sources of bias are inevitable.

## Conclusions and implications

Self-management behaviors play an important role in improving the prognosis and quality of life of patients with OSAHS. This study analyses the impact of general information, social support, and health literacy on the self-management behavior of OSAHS patients. Caregivers can create platforms to help OSAHS patients access, perceive and correctly use social support resources based on relevant predictors. They can also improve the way they communicate with patients, enhance their health literacy and try other interventions to further optimize patients' self-management behaviors.

## Data Availability

The datasets used and/or analyzed during the current study are available from the corresponding author upon reasonable request.
